# Access to health services among culturally and linguistically diverse populations in the Australian universal health care system: issues and challenges

**DOI:** 10.1186/s12889-022-13256-z

**Published:** 2022-05-03

**Authors:** Resham B. Khatri, Yibeltal Assefa

**Affiliations:** School of Public Health, the University of Queensland, Brisbane, Australia

**Keywords:** Culturally and linguistically diverse populations, Challenges, Access, Health services, Australia

## Abstract

**Background:**

About half of first- or second-generation Australians are born overseas, and one-in-five speak English as their second language at home which often are referred to as Culturally and Linguistically Diverse (CALD) populations. These people have varied health needs and face several barriers in accessing health services. Nevertheless, there are limited studies that synthesised these challenges. This study aimed to explore issues and challenges in accessing health services among CALD populations in Australia.

**Methods:**

We conducted a scoping review of the literature published from 1^st^ January 1970 to 30^th^ October 2021 in four databases: PubMed, Scopus, Embase, and the Web of Science. The search strategy was developed around CALD populations and the health services within the Australian context. We used Preferred Reporting Items for Systematic Reviews and Meta-Analysis guidelines for selection and Arksey and O’Malley framework for analysis of relevant articles. A narrative synthesis of data was conducted using inductive thematic analysis approach. Identified issues and challenges were described using an adapted socioecological model.

**Results:**

A total of 64 studies were included in the final review. Several challenges at various levels were identified to influence access to health services utilisation. Individual and family level challenges were related to interacting social and health conditions, poor health literacy, multimorbidity, diminishing healthy migrants’ effect. Community and organisational level challenges were acculturation leading to unhealthy food behaviours and lifestyles, language and communication problems, inadequate interpretation services, and poor cultural competency of providers. Finally, challenges at systems and policy levels included multiple structural disadvantages and vulnerabilities, inadequate health systems and services to address the needs of CALD populations.

**Conclusions:**

People from CALD backgrounds have multiple interacting social factors and diseases, low access to health services, and face challenges in the multilevel health and social systems. Health systems and services need to focus on treating multimorbidity through culturally appropriate health interventions that can effectively prevent and control diseases. Existing health services can be strengthened by ensuring multilingual health resources and onsite interpreters. Addressing structural challenges needs a holistic policy intervention such as improving social determinants of health (e.g., improving living and working conditions and reducing socioeconomic disparities) of CALD populations, which requires a high level political commitment.

**Supplementary Information:**

The online version contains supplementary material available at 10.1186/s12889-022-13256-z.

## Introduction

People who are born overseas and speak other than English are often referred to as Culturally and linguistically diverse (CALD) groups in Australia [1]. In 2016, half of the first or second-generation Australian parents were born overseas; one in five Australians did not speak English at home [2], and nearly two in five (37%) Australians aged > 65 years belonged to CALD population [3]. People from CALD backgrounds are heterogeneous groups, and include temporary migrants (e.g., international students or temporary work-skilled), refugees, and asylum seekers who usually settled through Humanitarian Support Programs (HSP), and permanent residents and citizens of migrant’s backgrounds [1, 4]. They have diverse cultures, languages, religions, social values, and migration trajectories. Among CALD populations, refugees and asylum seekers are the most vulnerable subgroups. People from CALD backgrounds can be categorised as: migrants coming through HSP from conflict-affected countries; migrants coming from Asia, Africa and Latin America; and from high-income countries [5]. Currently, migrations to Australia are the highest from the Asian continent, especially from China and India. Australia’s migration policy emphasises ‘regionalising’ migration within the country; thus, the proportion of CALD populations living in regional areas is increasing [6–8].

People from CALD backgrounds in Australia experience multiple social disadvantages and face challenges in health and health care needs [9]. In the early years of post-migration, CALD populations have relatively better physical health (often referred to as Healthy Migrant Effect) than their Australia-born counterparts [10]. They have low rates of all causes of mortality and potentially preventable hospitalisations [11]. However, CALD populations, especially refugees and asylum seekers, face challenges in the new settlement, including inadequate skills and communication for employment [12, 13]. People with low wealth status have a higher burden of diseases and illnesses; they must focus on managing day to day tasks such as working and living conditions rather than health care. For people from CALD backgrounds, this can be exacerbated due to inadequate job skills resulting in fewer employment opportunities [14]. In addition, they usually have sociocultural ties with their country of origin (e.g., food habits), while their growing children acculturation with the Australian lifestyle [15]. Current COVID-19 deaths in Australia revealed three-fold a higher mortality rate among CALD groups than the general population [16]. High rates of COVID-19 hospitalisation and deaths occurred among people with multiple forms of diseases [17]. The high rates of COVID-19 related deaths among these populations suggest they are particularly vulnerable [16]. This is just one example of why we need to address the disadvantages CALD populations face. Such deaths could be due to poor access to health services and severe COVID-19 cases requiring health services at hospitals.

Previous reviews on access to health services among CALD populations were focused on specific health issues of CALD populations [18–21]. However, there is a dearth of synthesised evidence of available literature on multiple dimensions of CALD populations. To address this gap in research, this study aimed to review issues and challenges in accessing health services among people from CALD backgrounds in Australia. The findings of this study could inform policy and programs for better access to health services and explore potential areas of future research on health and health services in Australia.

## Methods

### Context of migration and CALD populations in Australia

From World War II to 1970, there was an increase in European migrants coming to Australia as the immigration policy sought migrants from England and Europe [22]. From the mid-70s, the number of migrants from Asia began to increase and has continued over the last four decades [22]. Since the 1990s, the migration policies have become multicultural and included migrants from all regions and nationalities [23]. Currently, permanent migration in Australia occurs through two programs: Skilled Migration Program (for skilled and family migrants) and Humanitarian Program (for refugees and those in refugee-like situations) [24]. Both programs have annual quotas, and the first scheme has a controlled selection process and allows young and healthy persons to immigrate to Australia. In contrast, the later program has no such selection programs but has fixed quotas [6].

Among the top 10 migrants’ countries that came to Australia in 2020, seven were non-English speaking, and six were from South and Southeast Asia [25, 1, 26]. In 2021, the median age of overseas-born Australian was 44 years (compared to 34 years Australian borns), with the highest age of migrants from Europe (England- 58, and Italy-72 years) [25].

Since the late 1990s, there has been a growth in temporary migration and is not subject to quotas or caps by the Government, and this exclusively focuses on improving the short-term economic contribution [27]. Since 2013, there has been a three-fold increase of migrants on temporary visas for more than eight years in Australia. Temporary migrants in Australia have contributed to the national economy by working in essential sectors (e.g., health, agriculture), producing services and goods, and paying taxes and fees to universities [6]. However, welfare restrictions have contributed to temporary migrant workers’ economic and health insecurity because they have no access to income support if they lose their employment, and more vulnerable to underpayment, and are not included in the National Medicare Scheme, and COVID-19 has intensified these insecurities [28].

In 2016, 37.1% of Australia’s 295,324 frontline care workers (childcare, aged and disability and personal) were born overseas, up from 31.2% in 2011, and higher than the proportion of overseas-born workers in the total workforce (30.6%) [29]. This comprised of migrant care workers from non-English-speaking countries (28%), temporary visas (76%), from South Asia (35%) and females ( 85%) [29].

### Research design and framework

This study utilised a narrative synthesis of available evidence using a scoping review framework outlined by Arksey and O’Malley [30]. This framework has been previously used in health system and services research [31]. We incorporated the following phases: i) identifying the key research questions through an iterative review/discussion, ii) identifying the initial potential studies based on the discussion, iii) searching literature in major biomedical databases; iv) collating data, synthesising, and reporting of the findings, and v) discussion among experts and utilising their feedback as a required steps in knowledge translation part of a scoping review methodology.

### Search strategy and selection criteria

**Fig. 1:**
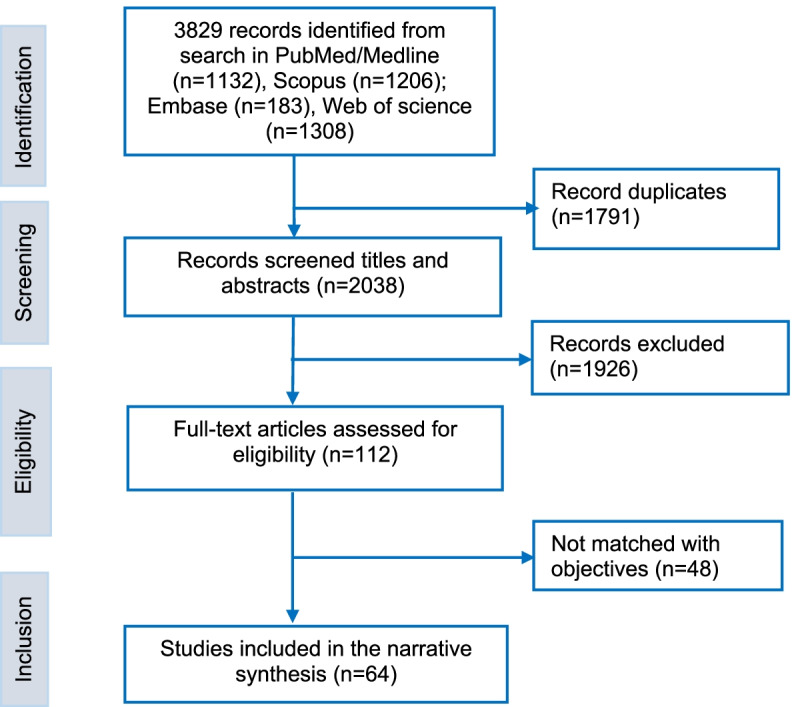
Articles selection process for the review

Four databases were searched: PubMed, Embase, Scopus, and the Web of Science. Search terms were identified and organised under three domains (supplementary material; Appendix 1): Health and health services and health systems; population groups; locations (Australia, states/territories). We included quantitative, qualitative, and mixed methods studies published in English from 1st January 1970 to 30th October 2021. We excluded study protocols and letters to the editors. The first author (RBK) developed the search strategy, and the second author (YA) reviewed and verified it independently. Then RBK searched records in databases, and assessed titles and abstracts of selected studies to evaluate their eligibility. Next, full-text studies were evaluated, discussed with the second author (YA). After consensus among the authors, studies were included in the final full-text review (Fig. 1). We presented this paper as a scoping review, following some components of the Preferred Reporting of Systematic Reviews and Meta-Analysis extension for Scoping Review (PRISMA-ScR) Checklist (see Supplementary material Appendix 2). The McGill Mixed Methods Appraisal Tool (MMAT) was used as a guiding framework to assess the quality of each study included in this scoping review [32]. We assessed the quality of the included studies in the context of our review's purpose, not in the context of the primary studies themselves and focused on the ability of the studies to answer our review questions.

### Data extraction and synthesis

Based on scoping review framework [30], themes on health issues and challenges and access to health services among people, CALD backgrounds were identified. We analysed data using an inductive approach and generated themes [33]. We adopted a multilevel socioecological model to explain and interpret the findings [34, 35]. This model helps explain the complex interplay between individual, relationship, community, and societal factors that influence health and diseases. For this study, we framed findings at the individual/family level issues that operate at the micro-level of the system, community and organisations challenges that operate and influence at the meso-level of the systems, and policy and systems-level challenges that influence at the macro-level [36]. At the individual and family level, people from CALD backgrounds experience social disadvantages, and high exposure to diseases that lead to multiple forms of diseases and illness. At the community and organisational level, lack of cultural competency, communication difficulties, cultural differences and lack of health awareness can influence the access and provision of health services. Finally, stigma and structural disadvantages can hinder access to health services among CALD groups and are systematic challenges.

## Results

Figure 1 shows the flow chart of the selected studies for this review. A total of 64 studies were included in the review.

### Multilevel issues and challenges of access to health services

**Fig. 2:**
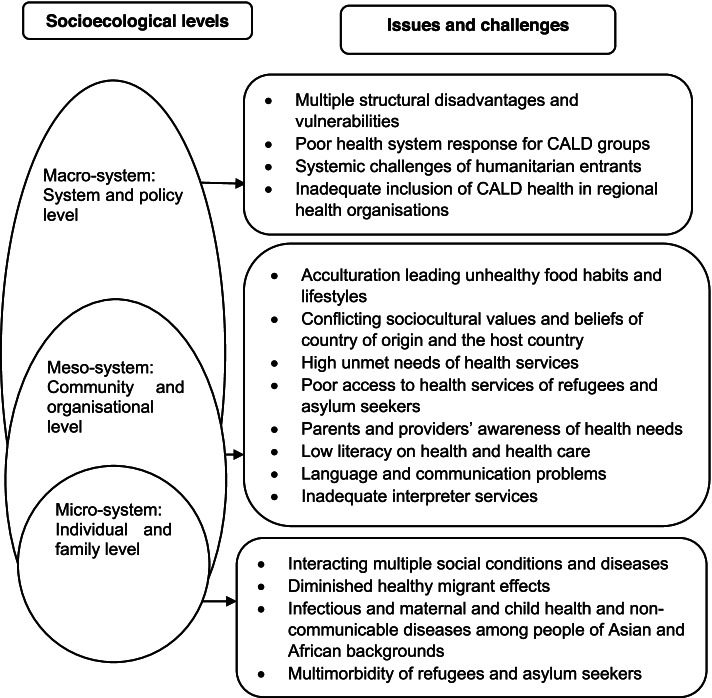
Multilevel issues and challenges on access to health services among CALD populations in Australia

Figure 2 describes the major challenges of accessing health services among CALD populations in Australia. Firstly, CALD groups experience problems at the individual and family level (e.g., multiple forms of interacting diseases including NCDs, infectious diseases, malnutrition). Secondly, they experienced challenges at the community and organisational level (e.g., change in lifestyle and food habits, low level of literacy and communication problems, supply of health services). Finally, people from CALD backgrounds face systemic challenges (e.g., structural disadvantages contribute to vulnerabilities).

#### Individual and family level issues and challenges

##### Interacting social conditions and diseases

Generally, CALD populations suffer from multiple diseases and illnesses (e.g., infectious diseases, nutritional and environmental problems, and NCDs). Among them, recently arrived CALD migrants had risky sexual behaviour and shared injecting drug equipment [37], while adult people of CALD backgrounds had a high risk of multiple chronic conditions (MCCs) (e.g., obesity and smoking, anxiety, depressed mood, and poor sleep) [38]. They had an increased risk of cardiovascular diseases (CVDs) [39], and children born from the parents of Middle Eastern backgrounds had a higher prevalence of overweight (53.0%) than non-CALD children (36.7%) in Victoria [40]. Among CALD populations of older age, females, born in CALD Middle Eastern countries had increased traumatic evidence, MCCs, and poor self-rated health [41].

##### Issues of CALD people of African descent: Infectious diseases, maternal and child health

The CALD women of African backgrounds had complex issues and health needs. For instance, people of Sub-Saharan African backgrounds had infectious diseases (e.g., chronic hepatitis B, schistosomiasis, and strongyloidiasis), female circumcision, and micronutrient deficiency (e.g., anaemia vitamin D, iron deficiency, thrombocytopenia) [42, 43]. In addition, women born in the East African countries had poor perinatal health outcomes; for example, women born in Sudan, Eritrea, Somali and Ethiopia had elevated odds of perinatal mortality, small gestational age, low birth weight, preterm birth, and increased obstetric complications [43, 44].

##### People of Asian backgrounds: Infectious diseases and risk of NCDs

Asian migrants had a high prevalence of chronic hepatitis B (CHB) than their African counterparts (54.3% vs 36.7%; *n* = 478); they were at risk of cirrhosis unless treated and increased cost over ten years [45]. Women born in Afghanistan, Bhutan, Iraq, and Myanmar, had poor maternal health, poor pregnancy care attendance, and late booking of visits [46]. People arriving through HSPs aged 35–44 years from Asia (e.g., Iraq and Middle Eastern countries) had higher triglycerides, hypertension, and smoking than their Australian-born counterparts [47].

##### Diminishing healthy migrant effects over time

First-generation CALD populations have better physical health than their Australian born counterparts. However, this advantage does not apply to mental health and diminishes once migrants spend some decades in Australia [48]. After a decade of stay in Australia, native-born Australians showed a clear health advantage over CALD groups [49]. In addition, there was an association between the length of stay and health and the gross domestic product (GDP) of the birth country, and the healthy migrant effect was negatively associated with CALD groups who were from low GDP countries than those from high GDP countries [50].

##### Multimorbidity of refugees and asylum seekers

Broadly, refugees and asylum seekers have multiple diseases and health needs. Evidence suggested that these people experienced several health challenges such as maternal and child health (MCH) and nutritional problems, higher exposure to risk factors of MCCs, and several infectious diseases. Refugees from Syria and Afghanistan had anaemia, teenage pregnancy, and a high prevalence of vitamin and micronutrient deficiencies [38, 46]. These CALD groups had more likely to have latent infection of tuberculosis, vitamin deficiencies, parasitic infections, and hepatitis B [51]. Additionally, they usually had a high burden of mental health disorders due to difficult migration journeys and conflict in the country of origin, which varied by generation and racial and ethnic disparities. A study reported nearly half of the study participants (48.8%) had probable post-traumatic stress disorder (PTSD) among refugees resettled in regional areas [52]. Another study reported that half of Melbourne's refugees had psychiatric disorders, while 22.9% and 31.3% of participants screened positive for PTSD symptoms in the previous month and lifetime, respectively [53]. Region of birth, age, high numbers of trauma events and living difficulties post-migration predicted depression and somatic symptoms among humanitarian entrants [54]. Anxiety, depression, and somatisation were higher among recently arrived refugee women from conflict-affected countries (e.g., Iraq, Lebanon, Sudan, and Burma) [54, 55], and recently arrived refugee women experienced high levels of psychological and financial distress, intimate partner violence [54–56].

#### Community and organisational level

##### Acculturation leading unhealthy food habits and lifestyles

In the early years of migration, they have good physical health; over time, migrants were exposed to risk factors of several NCDs (often referred to as the Exhaust Migrant Effect) due to access to a wide variety of food (e.g., takeaway food) and increased consumption of high energy take way foods and sedentary lifestyles that resulted in weight gain and obesity [57]. A higher proportion of Middle Eastern and South Asian children had consumed nutrient-poor snacks, transformed into family diets and physical activities, and sugar-sweetened beverages as takeaway foods [15, 40]. Length of stay and age of arrival also influence the development of risk factors, such as migrants from China aged < 18 years or who lived in Australia > 30 years were more likely to have diabetes and CVD risk factors [39].

##### Conflicting sociocultural values of the country of origin and host country

Some people of CALD backgrounds hold strong religio-cultural values and beliefs on health and illness of the country of origin. As a result, they struggled to adapt to new culture and health behaviours once they arrived in the host country. For instance, parents were strict with their values and culture, while their children preferred to eat high energy and processed foods [15]. Asian CALD communities were unwilling to access help from mainstream services because of their beliefs. Stigma and shame were key factors of reluctance and were not aligned with Western care approaches [58, 59]. South Asian women felt 'losing control over their pregnancy' because of their preoccupied diet and exercise to control their blood glucose levels [60]. Bhutanese migrants had cultural motivations and strong faith in doctors [61]. Women of African descent perceived concerns about the gender of the service providers, lack of privacy, and cultural and religious beliefs on screening of cancers [62].

##### High unmet needs of health services

People of CALD backgrounds had low utilisation of health services, experienced unmet healthcare needs, and faced barriers in accessing health services [63]. Women from Afghani backgrounds experienced negative side effects of hormone-based contraception. They expressed difficulty negotiating condom use with their husbands as an alternative, leading to inconsistent contraceptive practices and unintended pregnancy [64]. The new parents arriving with young children were not formally notified of MCH services, and there were disparities in service utilisation especially paediatric specialist services [63, 65]. Additionally, CALD groups had poor access to health services for chronic diseases, including mental health disorders. A study reported only 46.9% of migrants had sought professional help to mitigate mental health problems [52]. Women of South Asian backgrounds had not met their expectations in treating gestational diabetes practices [60].

##### Multiple barriers and poor utilisation of health services among refugees and asylum seekers

Newly arrived refugees and asylum seekers were faced with many difficulties in accessing effective health care. Prior to migration, refugees had complex health needs and experiences, and the challenges which contributed to social, financial, and psychological stress during resettlement in the host country [66, 67]. Humanitarian entrants had inadequate access to GP services and dental, mental, and maternity care [67, 68]. Women of refugees had a decrease in the first ANC visit at less than 16 weeks' gestation, inequalities in MCH services persist, and high unmet need for child health services [63, 69]. Refugees experienced poor access to PHC services, including preventive healthcare for cervical or breast cancer screening [66, 70]. These people generally had poor health than Australia born populations and had remained limited PHC services affecting their health conditions [67, 71].

Asylum seekers had poor access to PHC services due to Medicare ineligibility, health care costs, and experienced social, financial, and psychological stress [67]. Unaccompanied and separated children, and those in detention experienced additional challenges in accessing health care [68]. In addition, refugees from Afghan, Rohingya, and Sudanese backgrounds had low risk perceptions, a lack of information on routine services (e.g., immunisation) [72], lack of knowledge of the hepatitis B virus [73].

##### Providers and parent’s awareness of health needs

Health care providers (HCPs) recognise sexual and reproductive health (SRH) as a complex issue among CALD backgrounds that require unique skills to deliver optimal health care [74]. There were issues of health services among children of CALD groups that included parents’ awareness of availability of PHC services, their beliefs on an understanding of children's development and choices of service providers of proximity and continuity, purpose of visit, language spoken by the provider and experience of services [75]. South Asian women with gestational diabetes felt self-management information provided was inadequate and inappropriate to their needs [60].

##### Low literacy on health and health care

CALD people of Chinese backgrounds had poor health literacy, especially people who migrated at an older age, recent immigrants, and those with low educational levels [76]. Thus, inadequate health literacy was identified in most first-generation Chinese immigrants who had poorer self-rated health [76, 77]. Likewise, people of African backgrounds had poor health literacy and knowledge of health and diseases [78]. In addition, studies found a lack of knowledge about cervical cancer and pap smear and had low food, and health literacy among CALD groups [62, 79, 80], and those living in regional areas experienced difficulties searching and understanding health information and seeking the right services at the right time [81, 82].

Humanitarian entrants had poor health literacy that influenced their health care seeking, including poor knowledge of quality medicine [83]. They faced challenges in navigating health services due to a lack of necessary information [18, 82]. Displaced migrants found low knowledge of STIs and HIV [37]. Lack of information and low literacy among Bhutanese refugees’ care-seeking behaviours was associated with no symptoms-no check-up [61].

##### Language and communication problems

CALD groups of refugee backgrounds faced language and communication problems among people of Chinese backgrounds with limited English proficiency that increased the risk of chronic heart diseases and had difficulties navigating health care systems/resources, especially older, and those with poor proficiency in English [76, 77]. Misinformation and poor availability of multilingual health materials also influenced poor utilisation of health services and health screening in regional areas among CALD groups [81, 82]. Communication difficulties further challenged self-recognition and care-seeking of mental health problems among members of separated families [52]**.** In addition**,** Vietnamese people had access barriers influencing health-seeking behaviours, including language difficulties and lack of health information in their language [77, 83]. Sometimes language barriers created culturally unresponsive interactions and challenge accessing and utilising quality PHC services [79, 80, 84]. Poor understanding of English contributed to difficulties in making phone bookings for MCH services and care for infectious diseases [65, 73].

##### Poor cultural competency of health care providers

Poor cultural competency of healthcare providers (HCPs) was also a barrier to delivering health services among CALD populations, especially around knowledge and engagement and care provision. HCPs discussed the impact of accessing SRH care in women's country of origin and the influence of resettlement contexts [34]. GPs and pharmacists had poor competency in understanding service users' language, which influenced access to PHC services [85]. The health care provider’s language and cultural competence influenced medicines and pharmacy services utilisation among CALD population [86]. The gestational diabetes practices could not meet consumer expectations among women of South Asian backgrounds due to insufficient culturally appropriate care [60]. Furthermore, the role of reception staff and recording of the language and interpreter's needs was well defined but lacked effective systems to share the information with clinicians [87]. Providers' lack of cultural and spiritual awareness, culturally inappropriate and inadequate information influenced to the provision of mental health services [88].

##### Inadequate interpreter services

Accessing PHC services was influenced by a lack of interpreter services or low utilisation. Interpreter service was available, but provision was insufficient, especially in regional areas [81, 85]. For example, a study found that 40% of refugee women giving birth in Australia required interpreter services [69], and only 48% reported using the government-funded translating and interpreting service [87]. Lack of interpreter services and poor availability of multilingual health materials further challenged getting necessary information on health, diseases, and services [81, 88]. Current interpretation policy does not allow family members and relatives as interpreters. Thus, extra time was required to arrange interpreters because of the lack of onsite interpreters. In addition, minimal interpreting support for diagnostic services and emergencies (e.g., labour and delivery) was found major barriers to accessing health services [87, 89]. Low availability of interpreter services made it difficult in negotiating GPs services, especially among humanitarian entrants and asylum seekers [67].

#### Systems and policy level

##### Poor health system responses for CALD populations

CALD populations experienced barriers to accessibility (e.g., shortage and turnover of staff, distance, travel time, time constraints), acceptability (e.g., poor access to female-specific services, lack of privacy, cultural and religious beliefs), affordability (e.g., cost of services financial costs, high cost of services) and contextual factors (e.g., poor housing and unemployment) in regional areas [62, 65, 81, 83, 85]. They had poor knowledge and understanding of the health system [34, 53, 90, 91]. Current healthcare provisions were culturally inappropriate to African mental health patients, not considering the pre-existing cultural knowledge resulting in disempowerment and loss of autonomy [18, 88]. The health system lacked culturally appropriate service provision and recognition of sociocultural and religious during services delivery [78]. In the post-migration settlement phase, childhood obesity prevention program was influenced by targeted junk food advertising to children, and the lack of mandatory weight checks in schools [79].

##### Systematic challenges of humanitarian entrants and asylum seekers

There were several systemic challenges for humanitarian entrants and asylum seekers. For example, newly arrived refugees had had difficulty negotiating services and faced complexities in the new system and resettlement [67, 68]. In addition, studies reported that humanitarian entrants from Afghan, Rohingya, and South Sudanese backgrounds perceived challenges in the treatment of hepatitis B that included previous experiences, time constraints, divergent views about treatment decisions and perceived inadequate clinical support [73, 92].

Refugees with mental health had dual vulnerabilities: the stigmatisation associated with mental illnesses and the access and utilisation of services [58]. They faced social disadvantages such as financial hardships, unstable housing, discrimination, social exclusion, stigma from providers, and logistical difficulties that influenced their existing problems [41, 90, 93]. Refugees had a high prevalence of mental health issues in the initial years of settlement. They had health systems challenges in accessing mental health (e.g., fear of family members, being judged by treatment providers, fear of hospitalisation) [41, 90, 93]. Some refugees had a strong faith in health service providers, especially doctors; however, the health system lacked opportunistic screening for cancers during routine visits [61]. Nonetheless, they faced challenges from HCPs a lack of training knowledge resources and targeted services to address sexual and relationship issues [34, 71], and had poor accessibility to GPs services [66].

##### Inadequate inclusion of migrants and refugees’ health in regional organisations

Needs assessment documents of Medicare Locals (MLs) and Primary Health Networks (PHNs) identified that the health of CLAD populations included 46% of MLs and 74% of PHNs. But 48% of MLs and 55% of PHNs did not report any activities on migrant health, while 78% and 62% did not report any activities for refugees [94]. Factors associated with little attention for refugees and migrants’ health in MLs and PHNs included lack of local priority areas, funding, collaboration with the organisations working with CALD communities [94]. Moreover, current systems collect limited information about CALD groups (e.g., maternal country of birth, year of arrival in Australia, a requirement for an interpreter, and women’s preferred language) [95].

##### Multiple structural disadvantages and vulnerabilities

People from CALD backgrounds experienced several challenges at the micro-level from both supply and demand sides of the systems. However, these were the systemic challenges that influenced services delivery and utilisation. Their perceived challenges included stigma, embarrassment, fear, racism and discriminatory practices, poor knowledge and understanding of the health system, and difficulties in navigating the systems [53, 62, 78, 90, 91]. Those CALD groups with mental health issues experienced more vulnerabilities, including low self-esteem, lack of friends and relatives, poor understating of the health care systems [78, 84], shame and stigma, and lack of collaboration in health care [59]. Additionally, HIV related stigma and discrimination influenced non-disclosure, reduced social support, delayed testing and service access, and impacted treatment adherence [96]. Furthermore, they experienced embarrassment and difficulties adapting to a new cultural context [62, 81]. In addition, people from CALD groups faced financial stress, low socioeconomic status, poor housing, and unemployment, especially in regional areas [78, 81, 83]. Still, some migrants lacked social security services, including the Medicare scheme [88]. Moreover, high cost of care, long waiting time in case of MCCs, and the dissonance between providers and CALD services users in preference of alternative intentions [84, 91].

## Discussion

This review identified several challenges in accessing and utilising health services among CALD populations in Australia. These vulnerable groups face challenges in access to health services at individual and family level (e.g., interacting social conditions and diseases, infectious diseases, NCDs, and multimorbidity). At the community and organisational level, challenges include acculturation leading to an unhealthy lifestyle and food habits, diminishing healthy migrant effect, high unmet needs of health services, poor access to health services among humanitarian entrants, poor health literacy on health and health needs, language, and communication problems, and inadequate or low utilisation of interpreter services. Finally, system and policy level challenges include multiple structural disadvantages and vulnerabilities and inadequate inclusions of migrants’ health in regional public health organisations. Several strategies could be adopted to address these challenges at the multiple levels of the systems.

### Individual/family level strategies

Implementing tailored and context-specific program approaches and interventions can address issues and challenges at the individual level. People of CALD backgrounds have a higher risk of diseases and experience interacting adverse social conditions and multimorbidity. Targeted behaviour changes approaches can reduce potential risk factors of NCDs. Potential strategies include promoting a low-calorie diet and physical activity. Treatment of multimorbidity needs comprehensive assessment and treatment mechanisms than episodic treatment of disease. For instance, if any ill persons of CALD backgrounds visit GPs to treat common illnesses, GPs need to assess other associated disease conditions and illnesses and advise care seekers of any potential risk factors [97]. Previous background of individuals and country of origin is important as individuals’ belief and perceptions can affect the uptake of behaviour and daily practices [98]. Health and diseases conditions of CALD groups depend on their migration journey and their social-cultural backgrounds. Thus, individual program interventions need to focus on factors contributing to individuals’ social conditions and the progression of diseases. The aggravating causes of multiple diseases among CALD populations are interaction of diseases and social conditions [99]. Therefore, prevention and management of diseases among CALD groups need to have a holistic lens of social determinants of health.

### Community and organisational level strategies

Addressing individual/family level challenges also depend on community and organisational level strategies from both supply and demand perspectives. For instance, post-migration CALD populations generally suffer from many NCDs because of unhealthy food behaviours and sedentary lifestyles. Community factors, such as bicultural playgroups, ethnic community groups, and school-based healthy lunch box initiatives, can improve the reduction of childhood obesity, health literacy, and health promotion for NCDs [79]. Patients’ social networks and supports, religious beliefs and individual resilience were coping strategies, while common ethnic social support was positively associated with improved health problems [41, 92]. Family factors and social networks were positively influenced information-acquisition, health-seeking, and preventive behaviours [52].

Furthermore, improving health literacy and awareness of health, diseases, and needs are essential for behaviour changes. Studies reported that functional health literacy of younger-educated women improved care-seeking due to changing awareness [61] and creating a supportive health care environment (culturally responsive care, using trained interpreters) [80]. CALD parents had diverse experiences with service providers influenced by their awareness of available services during their stay in Australia [75]. Phone ownership indicated mHealth solutions acceptable to improve healthcare access, literacy, and autonomy among refugees in Melbourne [70]. Therefore, improving health literacy is vital for accessing health services among CALD populations.

Additionally, adequate, and timely language interpreter services could address language and communication barriers. Health education and communication resources materials need to be developed in participants' (service users') languages. A study reported that the implementation of reminder systems facilitated the uptake of health services and a range of GP practices [72]. The use of family members and relatives effectively interpreted GPs’ prescriptions [87], but they cannot act as interpreters. The feasibility study on the use of family members and friends as interpreters can ensure the provision of timely and uninterrupted interpretation services. Engaging bicultural workers and onsite interpreting services can improve trust and explain refugees' experiences [18, 71] and educate women on maternity care [43]. Community engagement can play a vital role in sharing of information. Studies reported that community organisations played a pivotal for disseminating culturally meaningful information on immunisation [72], and engaged refugee women in health screening programs and services [82].

Not only demand-side community factors, but provision of supply of health services also can improve the utilisation of health services. For instance, timely referral, awareness of risk factors, and appointment reminders can improve access to health services. Previous studies revealed that pre-arranged group appointments by MCH nurses increased engagement and participation with the health system and provided culturally appropriate services among newly arrived migrants [65, 100]. A study reported practice nurses’ supportive roles offer practical strategies for improving community knowledge about safe medicines [101]. Opportunistic screening (e.g., screening of breast and cervical cancers) in GPs clinics and advice about follow-up were found effective to identify multimorbidity [100]. Professional training and development of providers and the ability to recognise the role of medicine (e.g., mental health) enhanced health care-seeking and cultural responsiveness [43, 56].

Moreover, providers of similar backgrounds to service users can identify cultural variations and recognising such cultural differences is important for effective service provision that can address stigma and discrimination [78]. Such as recruiting health workers from CALD communities to adequately elicit and address patients' needs [91]. Furthermore, continuity of care nurses and interpreters from cross cultural groups were preferred for increasing client-provider trust and ongoing engagement in aged care facilities [65, 102]. Integrating CALD staff into the workforce can assist new migrants in transitioning into the wider Australian society and enriching the care of older persons [102]. The perception of gender roles and the involvement of male partners were integral to SRH decision-making and women-centred care [34]. Thus, the provision of culturally competent providers and cross-cultural health force is vital for delivering health services among CALD populations.

From both the demand and supply side of community and organisation level, humanitarian entrants and asylum seekers had multiple health problems and needs and poor access to health services, which suggests the need for targeted program interventions. Possible approaches could be providing friendly services during the first consultation visit, friendly staff, and connection with the refugees’ communities with similar social and ethnic backgrounds. Evidence revealed that these approaches were found effective for clinical assessments and prescriptions among Afghan refugees in Melbourne [68] and addressed their needs for family planning services [64]. Health providers need training on refugee health that could improve access to and quality use of medicines [101], and be able to provide services for women’s health (e.g., sexual and reproductive health) [74]. Refugee families established early connections with the community and religious groups with cultural, social their ethnic backgrounds [103]. The recruitment of cross cultural health workers is crucial to delivering services to this vulnerable subsection of CALD groups. Such health providers can provide culturally responsive care and improve the healthcare experience by providing people-centred care [34], and can build trust among users and providers [77]. Furthermore, the collection of basic information can help to address the health needs of humanitarian migrants and asylum seekers [95].

### System and policy level strategies

Macro-level strategies can address policy and systems-level challenges; however, they require political commitment and leadership. Structural interventions can influence the operationalisation of macro-policies at the organisational/ institutional level and implementation targeting the individual level. To address the policy and system level, a range of interventions can be adopted. For instance, the inclusion of CALD issues in the pre-service training or academic curriculum can be important for future HCPs for delivery of culturally appropriate services. Organisational ethics and values influence the human resource diversity management strategies and impact the quality of care provided to residents [102]. The current information system lacks important longitudinal health data of CALD population groups post migration in Australia. Ongoing monitoring of quality improvement initiatives needs such data on CALD groups to identify the vulnerable section of CALD groups and their health needs [69]. The inclusion of migrants' and refugees' health in PHNs and MLs is important for implementing public health programs.

Additionally, increasing the scope of the Medicare program can reduce the financial barriers of temporary migrants in accessing health services in Australia [28]. Low hostility towards migrants and refugees is a constant cultural force that can reduce such structural discrimination [102]. Rural resettlement of migrants was a mixture of settlement experiences and opportunity for integration, a sense of safety, and social connectedness [104]. Thus, integrated settlement can address the social discrimination and societal harmony of multicultural communities.

#### Implications for policy and research

This review has identified some program and research implications for CALD populations. Firstly, CALD populations often experience disadvantages and vulnerabilities leading to MCCs. Thus, health system efforts require addressing MCCs and their interacting social conditions. Secondly, there is a need to strengthen existing interpretation services by providing onsite timely interpreter services. Providers of the same ethnic backgrounds and multilingual education materials can improve access to health services. Thirdly, to address structural vulnerabilities, there is a need of policy interventions that can address systemic challenges. This research has identified some research agendas. Firstly, there are waves of migrations from different subcontinents and travel using different routes, have multimorbidity and health needs, health care experience and service utilisation and interacting social and diseases conditions. Exploring the status of multimorbidity and its social and cultural aspects is necessary, including the process of acculturation and behaviour changes. Secondly, there are interpretation services to address the language and communication barrier, but the use of these services is inadequate. Thus, family members and relatives can provide interpretation services timely and fill the need of inadequacy of interpretation services; this can be one of the research agendas to explore its feasibility and address language and communication barriers among CALD groups.

Thirdly, a significant proportion of CALD populations work in health care sectors, including aged, disability, and community care. However, access to health services among these care providers is inadequately explored; therefore, future research can address these research gaps. Fourthly, there are several cohorts of migrants settled in Australia. Longitudinal studies are required that can examine health problems which emerge in different cohorts across their lifecycle since arrival in Australia. Such a study can explore the health and diseases of CALD populations. Finally, CALD populations can face challenges in accessing health care at multiple stages, such as at the stage of seeking care and reaching care and receiving care at facilities. Therefore, further research is necessary that can explore delaying care at different stages and potential strategies to mitigate those delays for improved health services utilisation.

#### Strengths and limitations

This study systematically reviewed the available evidence on access to health services among CALD groups. The evidence was thematically synthesised and presented in a multilevel socioecological framework. The findings of this study could provide research, policy, and program insights to deliver the health services for the improved health status of CALD populations. Addressing access barriers is important to realise universal health coverage and achieve SDG3. Nonetheless, health services utilisation and delivery issues are complex and depend on health systems and social contexts. Therefore, findings from this study could signal which factors to consider and what levels of the system are vital for system performance. Identifying challenges could be the beginning steps toward further research agendas for access to health services for CALD groups in Australia. The CALD populations are heterogenous groups, and thematic synthesis provides important perspectives on issues and challenges in multilevel health systems in line with the research question; however, such analysis can miss the details of the individual study findings and issues of specific subsections of diverse groups.

## Conclusions

Despite the Australia’s national universal health care program (Medicare), some populations, such as people from CALD backgrounds, have poor access to quality health services. They experienced multiple challenges that could operate in multilevel health systems and had poor access to health services. Health services providers need to incorporate the care, prevention, and treatment mechanisms. Moreover, program approaches need to focus on preventing the risk behaviours of NCDs, and modification of lifestyle and unhealthy food behaviours. Program and policy efforts need to focus on vulnerable groups such as refugees and asylum seekers. Language and communication problems can be improved by strengthening existing interpretation services and ensuring multilingual information and communication materials. Recruiting health care providers of similar ethnic backgrounds can provide culturally appropriate care for a disadvantaged segment of CALD populations. High-level policy interventions can address the structural challenges, such as the provision of Medicare for CALD populations (e.g., temporary migrants) and the inclusion of migrants’ health in regional health organisations. Addressing structural challenges needs a holistic policy intervention such as improving social determinants of health (e.g., improving living and working conditions, improving socioeconomic status, reducing racial discrimination) of CALD populations, which requires a high level political commitment.

## Supplementary Information

**Additional file 1.**

## Data Availability

All relevant data are available within the paper and its supplementary material file.
